# Time Series Modeling of Tuberculosis Cases in India from 2017 to 2022 Based on the SARIMA-NNAR Hybrid Model

**DOI:** 10.1155/2023/5934552

**Published:** 2023-12-14

**Authors:** Baikunth Kumar Yadav, Sunil Kumar Srivastava, Ponnusamy Thillai Arasu, Pranveer Singh

**Affiliations:** ^1^Department of Zoology, Mahatma Gandhi Central University, Motihari 845401, Bihar, India; ^2^Department of Physics, Mahatma Gandhi Central University, Motihari 845401, Bihar, India; ^3^Department of Chemistry, College of Natural and Computational Sciences, Wollega University, Post Box No. 395, Nekemte, Ethiopia

## Abstract

Tuberculosis (TB) is still one of the severe progressive threats in developing countries. There are some limitations to social and economic development among developing nations. The present study forecasts the notified prevalence of TB based on seasonality and trend by applying the SARIMA-NNAR hybrid model. The NIKSHAY database repository provides monthly informed TB cases (2017 to 2022) in India. A time series model was constructed based on the seasonal autoregressive integrated moving averages (SARIMA), neural network autoregressive (NNAR), and, SARIM-NNAR hybrid models. These models were estimated with the help of the Bayesian information criterion (BIC) and Akaike information criterion (AIC). These models were established to compare the estimation. A total of 12,576,746 notified TB cases were reported over the years whereas the average case was observed as 174,677.02. The evaluating parameters values of RMSE, MAE, and MAPE for the hybrid model were found to be (13738.97), (10369.48), and (06.68). SARIMA model was (19104.38), (14304.15), and (09.45) and the NNAR were (11566.83), (9049.27), and (05.37), respectively. Therefore, the NNAR model performs better with time series data for fitting and forecasting compared to other models such as SARIMA as well as the hybrid model. The NNAR model indicated a suitable model for notified TB incidence forecasting. This model can be a good tool for future prediction. This will assist in devising a policy and strategizing for better prevention and control.

## 1. Introduction

Tuberculosis is a highly infectious disease that is the primary cause of ill health and one of the leading responsible factors that cause death throughout the globe [1]. Until the COVID-19 pandemic, TB is the foremost cause of death from the single most significant factor, HIV/AIDS [2]. TB is caused by bacillus *Mycobacterium tuberculosis*, which is transmitted from person to person through oral precipitations when an infected individual coughs or sneezes [3]. According to WHO health reports, the incidence rate of TB diminished from 7.1 million in 2019 to 5.8 million in 2020 [2], but the death rate increased in 2020, i.e. 1.3 million in 2020 whereas, 1.8 million TB deaths in 2019 occurred throughout the world [2]. In India, the situation is the opposite, i.e. the number of notified TB incidents reported in 2021 was 19,33,381, which was 19% higher than in 2020 (16,28,161) [4]. India faces the greater burden of TB incidence throughout the world with an estimated rate of 188 per 100,000 population (129–257 per 100,000 population) as of 2021 [4]. Although the worldwide TB incidence is showing a declining trend from 1% to 2% per year, it is still a significant global threat to public health, especially in developing countries [5].

Population gatherings, cultural programs, and religious festivals are the influencing factors that can increase TB's incidence rate during this time. It can help to monitor the trend of TB incidence and establish an accurate model to predict and control the further transmission of TB [6]. Several studies have been conducted in different geographical regions, such as India's eastern, western, northern, and southern regions [7, 8].

Time series analysis is a statistical technique that deals with time series data or trend analysis. It can determine the rate of infection among the population with various diseases such as tuberculosis, cancer, diabetes, and kidney disease. It can help to alleviate the burden of diseases by providing insights into the trends of diseases over time. It can help identify patterns and forecast future trends.

The ARIMA and SARIMA are the most suitable time series forecasting models proposed by Box and Jenkins 1970 [9]. The time series forecasting model completely depends upon the fitting values of previous data that explore future values. These models consist of 5 expressions such as AR (*P*), MA (*q*), ARMA (*p*, *q*), ARIMA (*p*, *d*, *q*), and ARIMA (*p*, *d*, *q*) (*P*, *D*, *Q*) s. This mathematical model is required to forecast TB occurrence, which provides an early warning system for the control of the disease. ARIMA and SARIMA models have frequently been used in forecasting epidemic or pandemic diseases such as COVID-19 [10–12], Malaria [13], Influenza [14], Hand foot & mouth disease [15], and as well as TB [16].

SARIMA model is widely utilized in the field of infectious diseases for future prediction [17, 18] and has also been adopted as a primary method throughout the world in TB incidence prediction [19]. However, the trend related to the impact of seasonality on TB cases nationwide has not been observed [8, 20]. A previous study conducted in India based on an assessment of seasonality and trend reported that the northern region has high seasonal variation while the southern and central regions have very little or no seasonality reported [21]. Some other countries have also reported seasonal variations such as China [22] and the United Kingdom [23]. Various seasonal factors such as precipitation, humidity, temperature, and day-night length vary according to the geographic regions, which might be responsible factors but their impacts are not completely understood. Therefore, with reference to TB, it is critical to analyze the seasonality to identify emerging concerns about TB. This may aid in devising future protective strategies for prevention and control [19]. Apart from that there are several studies conducted in previous years nationwide on TB prediction in other countries based on the SARIMA model that only reflects linear information [24, 25]. In this study, we used the SARIMA model that considers the linear information to assess the nationwide incidence of TB in India. However, the neural network autoregressive model takes into account the nonlinear information.

Artificial neural network (ANN) models are time-series forecasting methods that are constructed based on general mathematical models, which permit the nonlinear relationship among the response variables and their forecast variables [26]. Nonlinear mapping has a powerful ability to predict with high accuracy. A nonlinear autoregressive neural network (NARNN) is an effective model that has high fault tolerance properties in time series forecasting methods. In some studies, this effective model is regarded as a neural network autoregressive (NNAR) [15]. However, some studies have mentioned that a single ANN model may not be able to show any connection between linear and nonlinear models of the time-series prediction [27]. Previous studies conducted on combined models SARIMA and ANN showed high prediction accuracy and overcome the inadequacies of single models [28]. The SARIMA model is the best fit for linear relation whereas the ANN model is the best fit for nonlinear relation of the models [29]. The combination of both the models are utilized for better forecasting of the time series models.

The primary goal of the current study is to compare the time series forecasting efficiency of the SARIMA model, NNAR model, and the hybrid model with reference to the forecasting accuracy of the prevalence of TB cases in India. This study expects that the NNAR model has a better forecasting efficiency than the other time series models [30]. However, this model can help to predict future results on TB cases and facilitate better prevention control strategies as referenced information.

## 2. Materials and Methods

### 2.1. Data Collection

Secondary data on TB were extracted from the open repository web portal Ministry of Health and Family Welfare, Central TB Division, and Government of India (https://tbcindia.gov.in) from January 2017 to December 2022. All the secondary data were registered in the NIKSHAY repository (https://reports.nikshay.in/Reports/TBNotification) of notified TB incidences. We separately extracted data each month of every year, therefore, a total of 72 observations as a month over the 6^th^ year. This was followed by further incorporating the data into an Excel (2019) sheet to make a time series database. Time series analyses were accomplished in R-programming language (Vienna, Austria version 4.0.3) [31, 32] with integrated development for R in R-Studio (PBC, Boston, MA) [33] for making predictions, where (*P* < 0.001) was considered statistically significant.

### 2.2. SARIMA (1, 1, 2)(0, 0, 1) [12] Model

ARIMA is largely accepted and broadly used in time series forecasting methods for univariate analysis. Time series data with a seasonal component is known as the SARIMA model. This primarily consists of seasonal components (*P*, *D*, *Q*) and non-seasonal components (*p*, *d*, *q*) for interpretation of seasonality in a cyclic manner which repeats over the S period in the time series database. It is expressed as SARIMA (*p*, *d*, *q*) (P, *D*, Q) s where, *p* = AR order of nonseasonal, *d* = nonseasonal differencing, *q* = MA order of nonseasonal. *P* = AR order of seasonal, *D* = seasonal differencing, *Q* is the MA order of seasonal, and *S* = period of the repeated seasonal pattern.

The expression of the SARIMA model is given as follows[30]:

(1) $$\begin{array}{c} \phi \left(B^{s}\right)\varphi \left(B\right)\left(x_{t}-\mu \right)=\Theta \left(B^{s}\right)\theta {\left(B\right)}_{\varepsilon_{t}}. \end{array}$$

The nonseasonal components are as follows:

(2) $$\begin{array}{c} \text{AR}:\varphi \left(B\right)=1-\varphi_{1}B-\cdots -\varphi_{p}B^{p},\\ \text{MA}:\theta \left(B\right)=1-\theta_{1}B+\cdots +\theta_{q}B^{q}. \end{array}$$

The seasonal components are as follows:

(3) $$\begin{array}{c} \text{Seasonal AR}:\phi \left(B\right)=1-\phi_{1}B-\cdots -\phi_{P}B^{\text{PS}},\\ \text{Seasonal MR}:\Theta \left(B\right)=1-\Theta_{1}B+\cdots +\Theta_{Q}B^{\text{QS}}, \end{array}$$

whereas, *B* indicated a reverse shift, *ε*_*t*_ is a projected residual error at *t*, and *x*_*t*_ indicates an observed value at *t* (1, 2, . . . , *k*), *φ* is the route of the AR coefficient, *θ* is the route of MA coefficients, Θ is the route of seasonal MA coefficients, and Φ is the route of seasonal AR coefficients.

**Figure 1:**
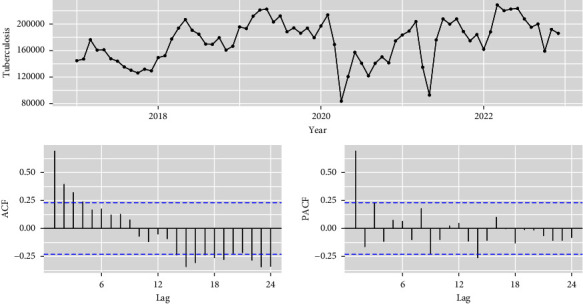
Time series with ACF and PACF plot of monthly notified TB cases from January 2017 to December 2022.

First, the stationarity of the time series data is essential for fitting the SARIMA model. Therefore, to check the data stationarity, an Augmented Dickey-Fuller (ADF) unit root test is applied. Seasonal and nonseasonal (*D* and *d*) differences are required to convert the nonstationary data into stationary data. Second, the order of the model was selected based on the autocorrelation function (ACF) and partial autocorrelation function (PACF) which is shown in Figure 1. After that, the lower values of the Bayesian information criterion (BIC) and Akaike information criterion (AIC) [34, 35] are required to select the appropriate model for the time series database of notified TB cases. Subsequently, we checked the reliability of the selected model, and its parameters were estimated then we used the model (*p*, *P*, *d*, *D*, *q*, *Q*) with its differenced values. ACF is associated with the past time-series TB data, whereas PACF is associated with the lagged values criterion of time-series data [36]. Both the BIC and AIC were penalized using and log-likelihood criterion. Finally, we checked the residuals with the help of ACF, PACF, and L-Jung Box tests. The residuals have hypothetically white noise with no autocorrelation among them.

Before applying the models, a time-series database of notified TB cases was split into 70^th^ and 30^th^ ratios of the training and testing datasets, respectively. The training dataset was selected from January 2017 to February 2021 (50^th^ observation) for training the models and the testing dataset was considered from March 2021 to December 2022 (22^th^ observation) for validation. All the training datasets were used for constructing the models and the validation dataset was used for forecasting performances of the models. The forecasting performance of the models was assessed by the following metrics: RMSE, MAE, and MAPE and finally, the models were used to forecast the prevalence of notified TB cases from January 2023 to December 2023.

### 2.3. NNAR (3, 1, 2) [12] Model

Artificial neural networks are the mathematical model of the brain, which is applied in the time series forecasting methods. It is widely used for complex nonlinear forecasting purposes [37]. With this model, lagged values can be used as an input for neural networks in the time series data and it is used as linear autoregressive models, and then the model is regarded as a neural network autoregressive (NNAR). This model is usually expressed as NNAR (*p*, *k*) and sometimes it is also considered as NNAR (*p*, *P*, *k*) *m*, whereas *p* is the lagged inputs, *P* is the seasonal lagged input, *k* is denoted as nodes in one hidden layer, and, *m* is the length of the seasonal period. An NNAR (*p*, 0) model is similar to an ARIMA (*p*, 0, 0) model, which is without any limitations on the parameters to confirm the stationarity. This model is also helpful for adding the last observation values in the same input of time series seasonal data. As an example, the NNAR (3, 1, 2) 12 model has inputs (*y*_*t*−1_, *y*_*t*−2_,…, *y*_*t*−3_) and *y*_*t*−12_ and with two hidden layers are in the neurons. NNAR (*p*, *P*, 0) *m* model is also similar to the SARIMA (*p*, 0, 0) (*P*, 0, 0) *m* model but there are no limitations on the parameters that make confirm the stationarity.

The general formations of these functions are defined as follows [37]:

(4) $$\begin{array}{c} {net}_{j}\underset{i}{\sum }w_{ij}y_{ij},\\ f\left(y\right)=\frac{1}{1+e^{-y}}. \end{array}$$

### 2.4. Hybrid (SARIMA-NNAR) Model

**Figure 2:**
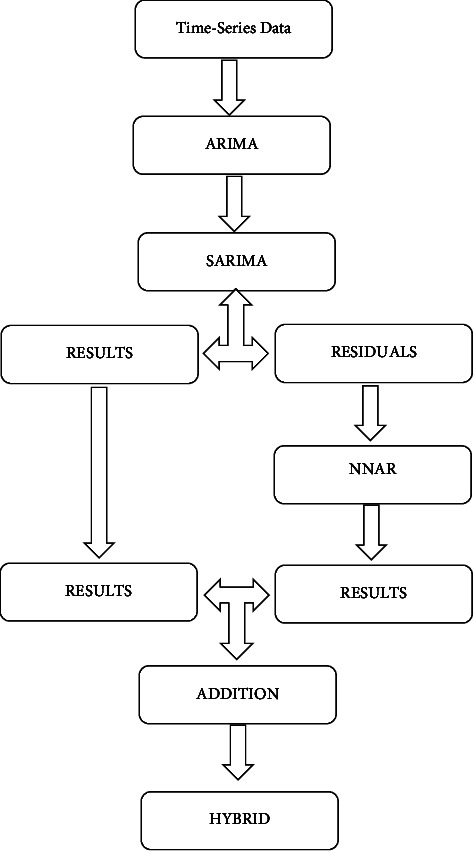
Structure of SARIMA-NNAR (hybrid) model.

A hybrid model was considered as it contains a linear and a nonlinear autocorrelation component. The SARIMA and the NNAR methodologies work together and predict the future values using observed past time series data and it is appropriate for nonlinear and linear issues, respectively. We also considered a hybrid model that combined the SARIMA model (linear) and NNAR (nonlinear) for the present study. The linear relations of the time series TB database were examined by using the SARIMA model while the residual part of the nonlinear relations has been examined by the hybrid mode [15, 38, 39]. Therefore, linear, and nonlinear sections of the hybrid model are combined and added for future prediction in this study. The linear part of the SARIMA and the nonlinear residual part of the hybrid models were selected to estimate the occurrence of TB cases at time *t*. The construction of the combined (SARIMA-NNAR) hybrid model is shown in Figure 2.

### 2.5. Forecast Assessment Methods

All the models were fitted in the past 72 months of the training data sets. The number of cases was forecast based on previous time series data. Among the SARIMA, NNAR, and hybrid models of forecasting results were evaluated with the assistance of four computational parameters. The four common methods to assess the results and outcomes in the forecasting models of time series data of TB cases are mean absolute error (MAE), mean absolute percentage error (MAPE), and root means square error (RMSE).

These three indices are expressed are given as follows [40]:

(5) $$\begin{array}{c} \text{MAE}=\frac{1}{n}\overset{n}{\underset{t=1}{\sum }}\left|x_{t}-{\hat{x}}_{t}\right|,\\ \text{MAPE}=\frac{1}{n}\overset{n}{\underset{t=1}{\sum }}\frac{\left|x_{t}-{\hat{x}}_{t}\right|}{x_{t}},\\ \text{RMSE}=\sqrt{\frac{1}{n}\overset{n}{\underset{t=1}{\sum }}\left(x_{t}-{\hat{x}}_{t}\right)}, \end{array}$$

here *x*_*t*_ is the definite occurrence, ${\hat{x}}_{t}$ is a projected occurrence, *n* is the forecast number, *A*_*t*_ is the definite value of the quantity being predicted, and *F*_*t*_ is a prediction value.

## 3. Results

**Figure 3:**
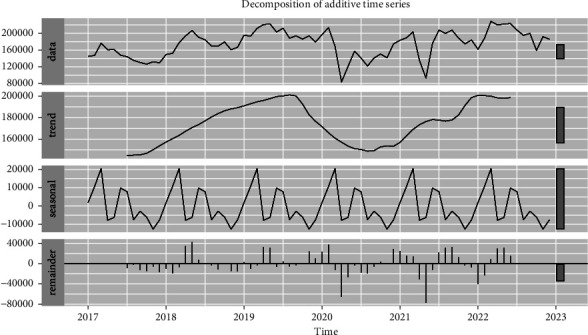
Additive decomposition of monthly notified time series plot.

The total notified TB incidences from January 2017 to December 2022 were reported at 1,25,76,746 cases over the years in India, given in Table 1. Descriptive statistics (mean, standard deviation, minimum, and maximum) were performed to the available databases where an average prevalence was (174677.02), standard deviation (31906.84), minimum (83647), and maximum (228814) values were observed over the years given in Table 2. The time series plot of the monthly occurrence of TB cases is depicted in Figure 1. The peak values of notified TB cases often occurred in March, April, and May. The additive decomposition function was used to diagnose time-series data of TB, which revealed the seasonal association that has cyclic changed every 12 months, shown in Figure 3. Peak seasons TB cases were seen in March, April, and May. Therefore, it has been confirmed that seasonal patterns are involved in seasonal indices of notified TB cases.

### 3.1. Performance in the SARIMA Model

ADF test was applied to the time-series nonstationary data (*P* value 0.3257) of notified TB cases. After one difference (*d* = 1) in the time series data, it became stationary (*P* value 0.05162). Due to this reason, we have selected the most appropriate model with the help of auto.arima() function [42]. The obtained model was SARIMA (1, 1, 2) (0, 0, 1) [12], having the lowest AIC-1632.85 whereas BIC-1646.43. It was the most appropriate model obtained in the R program by auto.arima() function of the forecast library [42–44]. Ljung–Box test was performed on this model to get a not significant *P* value is 0.3049 which indicated no autocorrelation in the residuals.

### 3.2. Performance in the NNAR Model

For this seasonal component, *P* was set to 1 for this seasonal component. The model that was automatically generated by the forecast library's nnetar () function [15, 40, 45]. We applied different *P* values multiple times and finally got the best prediction model (3, 1, 2) 12, which consists of the least error of the model. The obtained model established no autocorrelation in the residuals.

### 3.3. Performance in the Hybrid Model

The forecast hybrid package in the R program with the hybridModel () function [40, 45] was applied to determine a hybrid model. In this hybrid model, the SARIMA model is also determined by auto.arima () function, while the NNAR model is determined by nnetar () function. Both models worked together on training time series databases. The Ljung–Box test was performed and demonstrated no autocorrelation in residuals.

### 3.4. Accuracy Evaluation among the Models

**Figure 4:**
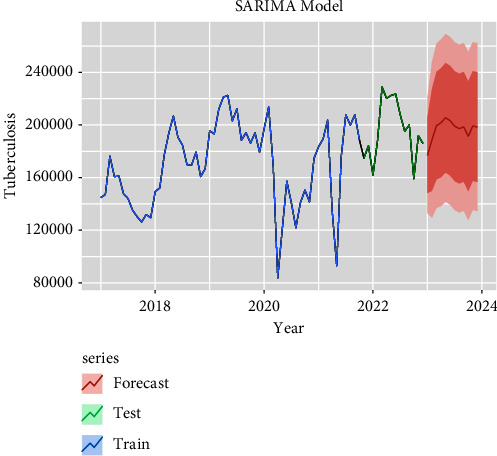
Forecast with ARIMA (1, 1, 2)(0, 0, 1) 12 model.

**Figure 5:**
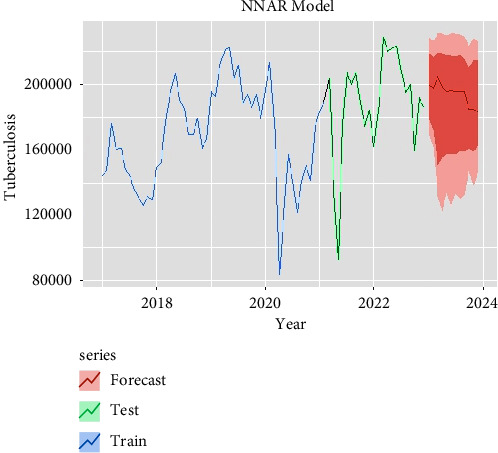
Forecast with NNAR (3, 1, 2) 12 model.

**Figure 6:**
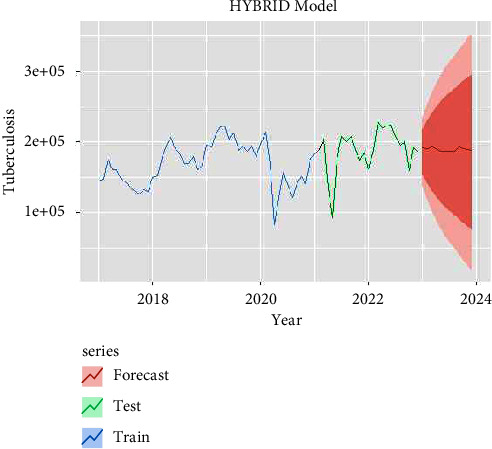
Forecast with hybrid (SARIMA-NNAR) model.

The performances of these three models (SARIMA, NNAR, and hybrid) in the training and testing datasets are shown in Table 3. All the criteria for comparison are mentioned in Table 3 which shows the RMSE, MAE, and MAPE values of hybrid and NNAR models having the least error values reported in the training dataset compared to SARIMA models. The forecasted TB transmission for the next 12 months is given in Table 4. The forecasting time series plots of SARIMA, NNAR, and hybrid models are shown in Figures 4–6 with training and testing data of notified TB incidences. The time-series models of forecasting values are approximately fitted to the actual notified cases of TB data which are depicted in Supplementary Figures 1–3. According to the models (SARIMA, NNAR, and Hybrid), it is seen that the number of TB notifications will remain constant in the coming months, with peak occurrences in March, April, and May.

## 4. Discussion

This study applied NNAR and hybrid models to estimate the number of TB notifications. The NNAR model was the best-fitted model for forecasting time series data of notified TB cases from 2017 to 2022 in India. To estimate the efficacy of the models, we compared the results of the models such as SARIMA, NNAR, and hybrid models. Both the models follow all the comparison criteria, which are required in time series data of TB cases. However, the best estimation results were obtained by the NNAR model followed by the hybrid model and SARIMA model, respectively. During the development of the NNAR model, there were various parameters utilized to forecast the best models. The hybrid model was also considered due to higher data characteristics in comparison to the nonhybrid model for forecasting [40]. The present analysis has suggested that the applied time series models, which showed the trend of TB due to seasonal variations might be a responsible factor that causes TB incidence in India, which shows periodicity in the notified TB database. This study also revealed an enhanced peak in the second quarter (March to May) and a decline in the fourth quarter (October to December) [20]. Several similar studies also show the seasonal influence in TB transmission [8, 38]. As per time series neural networks hybrid models, the projected results showed that the prevalence of TB cases in India would continuously increase in the coming months or years.

A similar study was conducted in provinces of China where SARIMA (0, 1, 1) (0, 1, 1) 12 and SARIMA-GRNN models predicted the prevalence of TB cases on the basis of influencing factors such as seasonality and trend [46, 47]. Another Chinese province has studied the prevalence of TB data by applying the SARIMA (1, 0, 0) (1, 0, 1) 12 model. These studies have suggested that the NNAR and hybrid model can efficiently explain the seasonality and trend in TB forecasting compared to SARIMA [48]. The basic origin model of an ARIMA has a widely accepted application model in the field of epidemic modeling or disease modeling. Further, it has extended to the SARIMA, NNAR, and different hybrid models.

However, the most suitable model was considered based on the results that are decided by the evaluation parameters, which are given in Table 2. NNAR is the first-best model, which was considered and the second-best model was the hybrid model for prediction. These models can help monitor notified TB incidences and help in adopting necessary measures accordingly.

The main strength of the current study is the analysis of nationwide data, which is the first concerning such studies in India. Also, for the first-time hybrid models along with SARIMA, and NNAR models are applied to nationwide TB data. However, the current study has a few limitations as well. Foremost there is a lack of demographic indexes such as age, gender, educational status, caste, and religion in the study. In addition, socioeconomic and climatic factors were not included in the study that could influence the seasonal variations. Finally, the opted models were derived using the data only from January 2017 to December 2022 in India and tested against only one year of the available datasets. Hence, the findings should be reassessed cautiously with the additional time-series data in future studies.

## 5. Conclusion

The finding of the study is based on time-series SARIMA, NNAR, and a hybrid model fitted on notified TB cases from January 2017 to December 2022 in India. We have estimated TB incidences by the NNAR and hybrid models. The NNAR model performs better than the individual SARIMA or the hybrid (SARIMA-NNAR) for predicting the prevalence of notified TB cases. This forecast will assist policymakers in devising better policies and strategies for the control and prevention of TB.

## Figures and Tables

**Table 1:** Monthly reported notified TB cases from January 2017 to December 2022.

Months	2017	2018	2019	2020	2021	2022
January	144781	149393	195596	196997	183398	161898
February	147133	152119	193142	213699	189377	188081
March	176283	177442	211868	169171	203648	228814
April	160671	193912	221100	83647^∗^	134825	220166
May	161146	206750	222455	120737	92827^∗^	222469
June	147705	190644	203098	157328	176007	223496
July	144041	184647	212255	140868	207751	207715
August	135239	169828	188278	121820	199885	195197
September	130291	169394	194154	140813	207685	200045
October	126237	179379	186196	150480	188803	159075
November	131882	160726	193955	141548	174757	191940
December	129434	166612	179260	174598	184173	185962

^*∗*^According to the WHO, the COVID-19 pandemic has had a significant impact on TB diagnosis and treatment in India. The ensuing disruptions from the health crisis arising out of the COVID pandemic have prompted lags in diagnosis and the beginning of treatment, which has resulted in a 25% annual shortfall in TB case notifications in 2020 compared with 2019 [41]. In addition, the COVID pandemic has led to the absolute mobilization of healthcare efforts and infrastructure towards its control and management, leaving no attention to other afflictions such as TB and others.

**Table 2:** Descriptive statistics for the available database from 2017 to 22 in India.

Observation	Mean	Standard deviation	Minimum	Maximum
2017	144570.25	15295.81	126237	176283
2018	175070.50	17162.71	149393	206750
2019	200113.08	13964.77	179260	222455
2020	150975.50	35128.03	83647	213699
2021	178594.66	33500.67	92827	207751
2022	198738.16	23136.01	159075	228814
2017−22	174677.02	31906.84	83647	228814

**Table 3:** Accuracy parameters of SARIMA, NNAR, and hybrid models of training and testing data set.

Models	Training set			Testing set		
	RMSE	MAE	MAPE	RMSE	MAE	MAPE
ARIMA (1, 1, 2)(0, 0, 1) [12]	19104.38	14304.15	09.45	32303.49	22797.31	16.69
NNAR (3, 1, 2) [12]	11566.83	9049.27	05.37	43777.35	36489.51	23.94
Hybrid (SARIMA-NNAR)	13738.97	10369.48	06.68	31591.70	21943.03	15.93

**Table 4:** Forecasting comparison between the models for January 2023 to December 2023.

Forecasts of notified TB incidence			
Time	SARIMA	NNAR	Hybrid
January 2023	176878.2	200034.5	192872.9
February 2023	188667.6	197658.7	192502.6
March 2023	199438.5	204757.0	193885.6
April 2023	201697.6	198512.6	191584.4
May 2023	205442.2	195662.8	189836.9
June 2023	203230.1	196040.8	189489.9
July 2023	199050.8	195976.4	191182.5
August 2023	197194.6	195673.3	191869.6
September 2023	198508.4	195615.5	191986.8
October 2023	191409.5	185075.7	187780.8
November 2023	199169.2	184996.2	190227.5
December 2023	198303.9	183353.1	191394.0

## Data Availability

The Secondary data were extracted from the NIKSHAY open repository web-portal; National Tuberculosis Elimination Programme (NTEP) (https://reports.nikshay.in/Reports/TBNotification). The datasets of notified TB cases used for analysis in the study from 2017 to 2022 were obtained from the Central TB Division, Government of India, and Ministry of Health and Family Welfare (https://tbcindia.gov.in).

## Abbreviations

AIC: Akaike information criterion
ACF: Autocorrelation function
BIC: Bayesian information criterion
MAE: Mean absolute error
MAPE: Mean absolute percentage error
NNAR: Neural network autoregressive
PACF: Partial autocorrelation function
RMSE: Root mean square error
SARIMA: Seasonal autoregressive integrated moving averages.

## Supplementary Materials

Supplementary Materials Supplementary 1. Supplementary Figure 1: Depicted actual, fitted, and forecasting values by SARIMA model in time-series plot. Supplementary 2. Supplementary Figure 2: Depicted actual, fitted, and forecasting values by NNAR model in time-series plot. Supplementary 3. Supplementary Figure 3: Depicted actual, fitted, and forecasting values by hybrid model in time-series plot.
