# Supplementary figures and images for: Time Series Modeling of Tuberculosis Cases in India from 2017 to 2022 Based on the SARIMA-NNAR Hybrid Model
Source: Can J Infect Dis Med Microbiol. 2023 Dec 14;2023:5934552. doi: 10.1155/2023/5934552 (PMC10748728; doi:10.1155/2023/5934552)

## NNAR Model

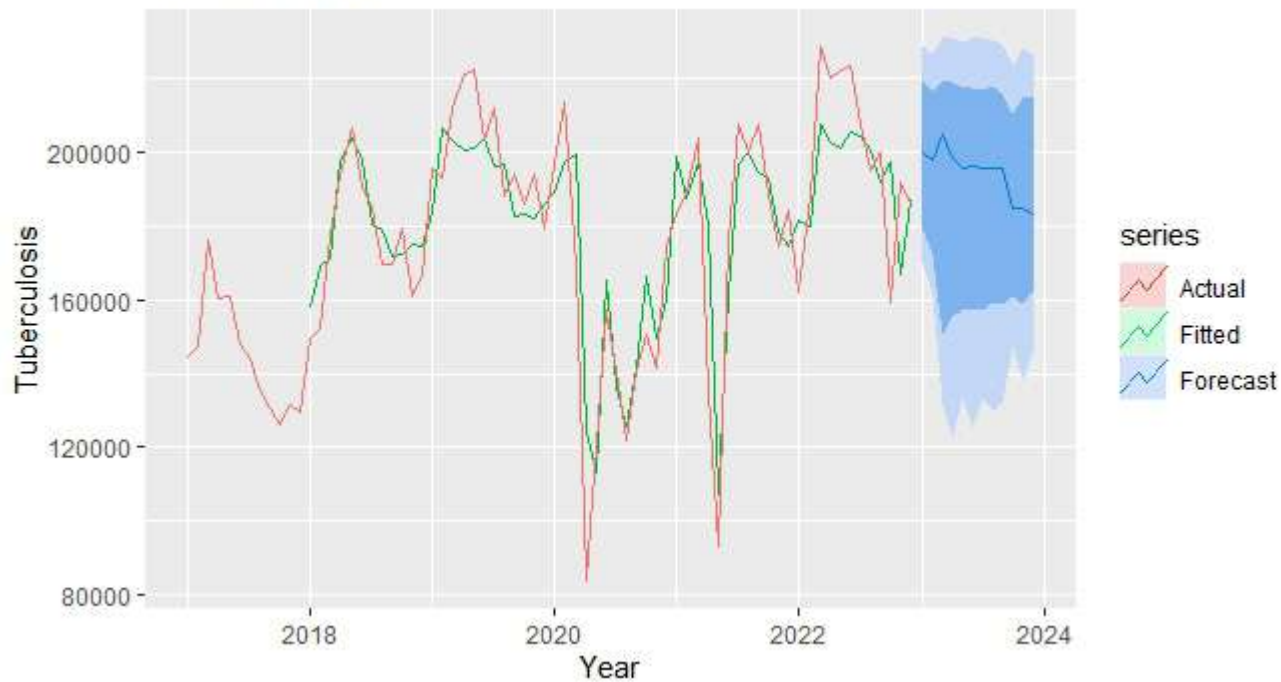

Supplement: Supplementary Materials — Supplementary 1. Supplementary Figure 1: Depicted actual, fitted, and forecasting values by SARIMA model in time-series plot. Supplementary 2. Supplementary Figure 2: Depicted actual, fitted, and forecasting values by NNAR model in time-series plot. Supplementary 3. Supplementary Figure 3: Depicted actual, fitted, and forecasting values by hybrid model in time-series plot. [file 5934552.f1.zip › SF1.pdf]

## SARIMA Model

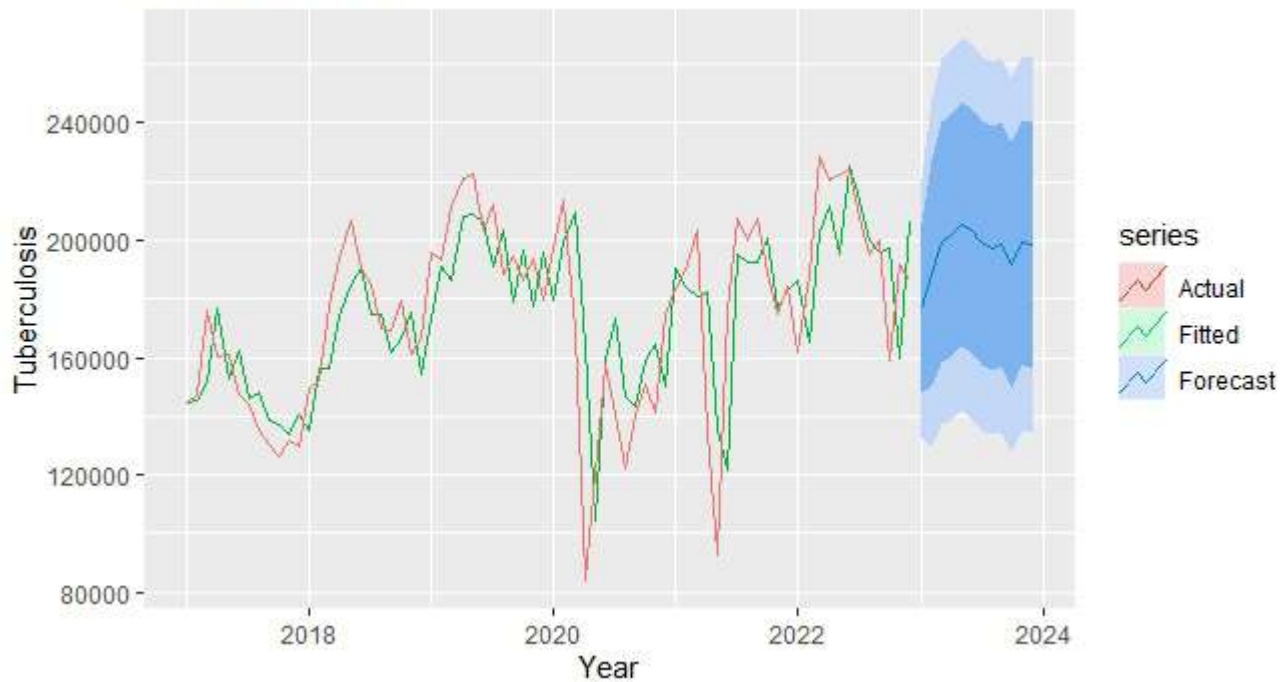

Supplement: Supplementary Materials — Supplementary 1. Supplementary Figure 1: Depicted actual, fitted, and forecasting values by SARIMA model in time-series plot. Supplementary 2. Supplementary Figure 2: Depicted actual, fitted, and forecasting values by NNAR model in time-series plot. Supplementary 3. Supplementary Figure 3: Depicted actual, fitted, and forecasting values by hybrid model in time-series plot. [file 5934552.f1.zip › SF2.pdf]

## HYBRID Model

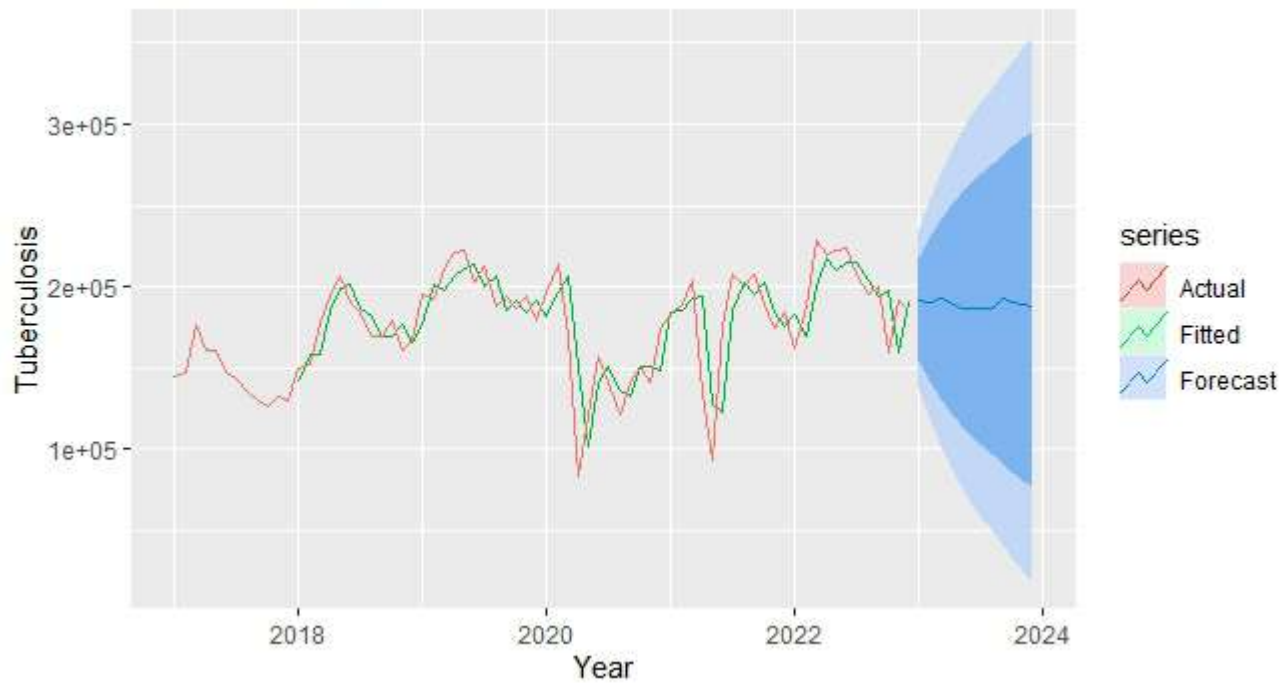

Supplement: Supplementary Materials — Supplementary 1. Supplementary Figure 1: Depicted actual, fitted, and forecasting values by SARIMA model in time-series plot. Supplementary 2. Supplementary Figure 2: Depicted actual, fitted, and forecasting values by NNAR model in time-series plot. Supplementary 3. Supplementary Figure 3: Depicted actual, fitted, and forecasting values by hybrid model in time-series plot. [file 5934552.f1.zip › SF3.pdf]
